# Applied Behavior Analysis as Treatment for Autism Spectrum Disorders: Topic Modeling and Linguistic Analysis of Reddit Posts

**DOI:** 10.3389/fresc.2021.682533

**Published:** 2022-01-05

**Authors:** Monica L. Bellon-Harn, Ryan L. Boyd, Vinaya Manchaiah

**Affiliations:** ^1^Department of Communication Sciences and Disorders, Piedmont University, Demorest, GA, United States; ^2^Virtual Hearing Lab, Collaborative Initiative Between Lamar University and University of Pretoria, Beaumont, TX, United States; ^3^Department of Speech and Hearing, School of Allied Health Sciences, Manipal, India; ^4^Department of Psychology, Lancaster University, Lancaster, United Kingdom; ^5^Security Lancaster, Lancaster University, Lancaster, United Kingdom; ^6^Data Science Institute, Lancaster University, Lancaster, United Kingdom; ^7^Department of Speech-Language Pathology and Audiology, University of Pretoria, Pretoria, South Africa; ^8^Department of Speech and Hearing Sciences, Lamar University, Beaumont, TX, United States

**Keywords:** autism spectrum disorder, Reddit, applied behavioral analysis, health management, topic modeling

## Abstract

**Background:** It is critical for professionals to understand the discourse landscape within various online and social media outlets in order to support families of children with autism in treatment decision-making. This need is heightened when considering treatments that have garnered excitement and controversy, such as applied behavioral analysis (ABA) therapy.

**Method:** The specific aims of this study were to identify the main themes in Reddit posts about ABA-based interventions for autism using topic modeling, to examine the linguistic aspects of Reddit conversations using the Linguistic Inquiry and Word Count (LIWC) analysis, and to examine the relationship between linguistic aspects and user category (i.e., pro- vs. anti-ABA vs. undecided, parent vs. professional vs. an individual with autism).

**Results:** The topic modeling resulted in 11 themes that ranged across various elements, such as autism as a condition and its management, stakeholders, and consequences of autism and the support needed. The posts of individuals were focused on personal experiences and opinions as opposed to clinical and research information sharing. Linguistic analysis indicated that the posts reveal an intimate stance rather than an empirical stance.

**Conclusions:** Results provide insight into perspectives of ABA. This type of research may help in developing and distributing appropriate and evidence-based information.

## Introduction

Families of children with autism spectrum disorder (ASD) must decide among varied types of management and intervention options to address symptoms associated with ASD, such as severe and sustained impairment in communication and social interaction and restricted patterns of ritualistic and stereotyped behaviors (1). Some children with ASD also exhibit difficulty in adaptive behaviors, psychiatric symptoms, and intellectual disability (2, 3). Families often turn to online information and other social media platforms for treatment decision-making guidance. However, information for families with children with ASD is frequently confusing and unreliable (4, 5). Further, social media platforms serve different functions for different stakeholders associated with ASD, which can influence the content and purpose of information. For example, Bellon-Harn et al. (6) reported that a number of Twitter users posting ASD-related tweets were associated with advocacy communities as compared to clinical and research communities. It is critical for professionals to understand the discourse landscape within various online and social media outlets in order to support families in treatment decision-making. This need is heightened when considering treatments that have garnered excitement and controversy, such as interventions based on principles of applied behavioral analysis (ABA) (7).

### ABA-Based Interventions

Applied behavioral analysis is science on which ABA-based interventions have been developed. ABA is derived from tenants of behaviorism, experimental analysis of behavior, and applied research, and its methods can be applied to a variety of intervention approaches for children with ASD (8). Evidence-based research is emerging; however, the consensus from meta-analysis studies is that more research is necessary to understand the efficacy and effectiveness associated with ABA-based intervention (9–11). More evidence may also clarify misinformation and diminish misuse surrounding the practice of ABA-based interventions (8, 12, 13).

In light of potential misconceptions about ABA, it is valuable to understand the content of information that is shared online and the sentiment of the content. Since individuals with ASD, family members, and other stakeholders utilize online communities (14), research examining online content provides an opportunity to learn about the experiences and voices of adults with ASD and other members of neurodiverse communities (15, 16). This may provide valuable information in understanding factors linked to decision-making related to ABA-based intervention. In turn, this may facilitate the ability of healthcare professionals to provide guidance to families on making informed choices based on evidence with a clear understanding of the benefits and limitations of their options (17). This study is an initial step to understand the discourse landscape surrounding ABA-based interventions for children with ASD within a social media platform. Specifically, we used topic modeling and linguistic analysis methods to examine ABA-related posts in Reddit.

### Reddit and ASD

Reddit (http://www.reddit.com/) is a social network that has many elements common in other popular social media sites (e.g., Facebook and Twitter), such as the ability to communicate and share information with other users, the ability to follow users and groups, and the ability to create one's own information. However, it is distinguished because Reddit's content is accessible to anyone with or without an account, and people can have “throwaway” accounts (i.e., temporary identities). Most Reddit users subscribe to more subreddits, which are defined as a smaller community of posters within a broader community of posters.

Some explorations of content and linguistic attributes within social media platforms, such as Reddit, have occurred. Types of analysis to examine large corpora of data include and topic modeling Linguistic Inquiry and Word Count (LIWC). Topic modeling is a technique that involves text-mining algorithms to identify patterns within the data (18). This method examines how words cluster together in their use. LIWC is an automatic text analysis program that counts and calculates the percentage of words in the text that match various emotional, cognitive, structural, and process dimensions. The LIWC program includes a main text analysis module, along with a group of built-in dictionaries. The text analysis module compares each word in the text against a user-defined dictionary (19).

Some analyses of Reddit corpora within the area of ASD are completed. For example, Thin et al. (20) examined conversational involvement, emotion, and informational support in a subreddit *r/Aspergers* using cluster analysis. Results indicate that the ASD subreddit was a supportive community. Saha and Agarwal (21) examined the social support of popular ASD bloggers active in blogs and Twitter LIWC analysis (19). Results indicate that the ASD community provides significant social support to its members both on Twitter and blogs. Bellon-Harn et al. (6) examined patterns and themes of ASD-related tweet content on Twitter. The authors reported that the language appears to be associated with a more guarded, distanced form of discourse rather than a personal form of discourse. The authors suggested the length of the tweet does not allow room for more personal forms of discourse, which may require more space to articulate the depth of thought.

### Summary and Study Purpose

This paper seeks to contribute to information centered on understanding the role of social media within the area of ASD. The specific aims include (a) to identify the main themes in online discussions around ABA-based interventions for ASD using topic modeling, (b) to examine the linguistic aspects of conversations using the LIWC analysis, and (c) to examine the relationship between linguistic aspects and user category (i.e., pro- vs. anti-ABA vs. undecided, parent vs. professional vs. an individual with ASD).

## Materials and Methods

### Study Design and Ethical Considerations

The study used a cross-sectional design. Conversations about ABA in relation to ASD were extracted from Reddit. No ethical approval is required as the data were anonymous, and no personally identifiable information was included (22). This was an analysis of public data, and the authors were careful to ensure analyses did not compromise user identity.

### Data Extraction

The data for this study consist of original posts (i.e., a submission that starts a conversation) and associated comments (i.e., a submission that replies to posts or other comments) from several topical focused subreddits (i.e., subcommunities). Two sets of data (i.e., discussions about ABA for autism and Reddit baseline data) were extracted *via* the Reddit application programming interface (API) using a custom-built script. The approach to data extraction was to collect the entire thread history. Where possible, all original post-level information was retained. In cases where comments remained but user-level information had been removed, all data were retained that was still available. This Reddit API is publicly accessible and allows researchers to acquire language data directly from the site without using the typical web interface. Reddit does not collect thorough demographic data on the users of the site, so we cannot describe the characteristics of the sample. The data posted from the time Reddit started through March 2020 were extracted chronologically.

To identify the relevant threads containing posts about ABA in relation to ASD, a search was performed in Reddit using the keywords “Applied Behavior Analysis,” “ABA Therapy,” “Autism,” or “Autism Spectrum Disorder.” These keywords were compiled based on consensus between researchers following searches in Reddit and Google trends (www.google.com/trends), a website that analyzes the popularity of search terms and uses graphs to compare the search volume of the terms over time. The search was sorted by relevance from all time, and the threads that had a focus on ABA were included. Although the data were extracted from 19 subreddit threads, most of the data were generated from a few threads, such as r/autism (62%), r/aspergers (13%), r/BehaviorAnalysis (6%), r/ABA (3.5%), r/Parenting (3%), r/unpopularopinion (2.7%), and r/IAmA (2.3%). A total of 2,432 posts were extracted. However, 112 posts were not relevant to ABA and were fewer than five words. As such, they were excluded. The remaining 2,320 posts were included for further analysis.

For the purpose of linguistic analysis, another dataset with baseline Reddit data was generated. For LIWC, the software provides output (results) on the percent occurrence for each of the psychologically meaningful dimensions. However, we do not know if this percentage is appropriate unless it is compared to a standard or baseline. We decided the best procedure was to examine Reddit data related to ABA-based intervention in comparison to other general Reddit conversations with data. Consequently, baseline data were generated. A subsample of 0.1% was extracted randomly from r/AskReddit, which resulted in a sample of 357,795 posts. Of these, 84,215 posts that had five words or less were excluded, and the remaining 273,580 posts formulated the baseline data corpus.

### Data Analysis

#### Category Determination

All posts were coded according to the view toward ABA and the personal identification of their status. Preliminary coding of the initially posted 100 posts provided the codes for whether or not the post (1) included support of ABA (i.e., pro-ABA); (2) include support ABA (anti-ABA); (3) was seeking information about ABA (i.e., neutral/curious); or (4) was not directly related to ABA (i.e., unclassified). Unclassified posts included posts giving feedback about what is or is not appropriate to post, another related ASD issue (e.g., diagnosis), or commenting on the relative value of a post. Following cyclical review by the first author and two graduate students in speech-language pathology, codes were developed. Pro-ABA codes were defined as posts that described ABA as beneficial and/or included a positive impact of ABA. Anti-ABA codes were defined as posts that described ABA as not beneficial and/or included a negative impact of ABA. Neutral/curious codes were seeking information about ABA or wanted to understand characteristics of ABA. Unclassified posts did not relate to ABA even though they were related to some aspect of autism causes, characteristics, or treatment.

Additionally, the posts were coded according to their personal identification of their status as a person with ASD, a parent of a child with ASD, a professional, or other. In order to be coded, the post explicitly stated their status (e.g., as an autistic adult). Upon review and discussion by the three coders, one graduate student completed coding the complete data set of 2,320 posts. Following each set of 100 posts, the sample was sent to the first author for review, consensus, and to resolve queries until all 2,320 posts were reviewed.

#### Topic Modeling

In this study, topic modeling was performed on all 2,320 posts using the Leximancer software (edition 4.0) (https://info.leximancer.com/) to identify the main themes, concepts, and their relationships within the posts. The use of Leximancer to derive semantic content and relationships from natural language, in this case written discourse, has been validated (23). This method uses a suite of algorithms to identify themes, concepts, and relationships resulting in an output that includes graphic summaries. The process of topic modeling involves (1) concept identification in which single, frequently occurring words are determined; (2) concept definition in which a group of words that form a concept is compiled; and (3) text classification in which the concepts that were identified and defined are analyzed for frequency of occurrence (18). Based on the output, insights into the nature of a particular discourse topic can be drawn (24).

The LIWC software program (https://liwc.wpengine.com/) was used to analyze linguistic aspects of the text data. In the current study, the research team identified 10 key linguistic variables, which were included for further analysis using LIWC. All texts with fewer than five words were excluded to prevent skew [see (25)]. For example, a post with a single word “Wonderful!” may result in a positive emotion score of 100%, which is not in line with the typical percentage (4–5%) for this category. Such a cutoff is a common convention when performing LIWC (25). The LIWC has high internal reliability and external validity and is validated across thousands of studies (19, 26).

#### Statistical Analysis

SPSS software was used for statistical analyses. The assumptions of normality and the assumption of homogeneity of variance were tested using the Shapiro-Wilks test and Levene's test, respectively. As the data met these assumptions, parametric statistics were selected. A one-sample *t*-test was performed to compare the linguistic variable results with the baseline Reddit data. One-way ANOVA was used to test for differences in language use between user categories. A *p*-value of 0.05 was used for statistical significance interpretations.

## Results

### Review Characteristics

Of the 2,320 Reddit posts, 2,140 came from unique users. Of these, 75 were original posts; the remaining 2,245 were comments. For the original posts, the median up-vote ratio was 0.9, and the median ups were seven suggesting that these posts were quite popular on the Reddit platforms. Table 1 shows the user categories of these posts based on the view of users toward ABA and their relationship.

**Table 1:** Reddit user categories.

**User categories**	***N* (2,320)**	**%**
**View toward ABA**		
• Pro ABA • Anti ABA • Undecided (or curious) • Other (unable to categorize)	493 481 201 1,145	21.3 20.7 8.7 49.3
**Personal status**		
• Parent • Professional• Individual with ASD • Other	366 296 317 1,341	15.8 12.8 13.7 57.6

### Topic Modeling: Themes and Concepts

**Figure 1:**
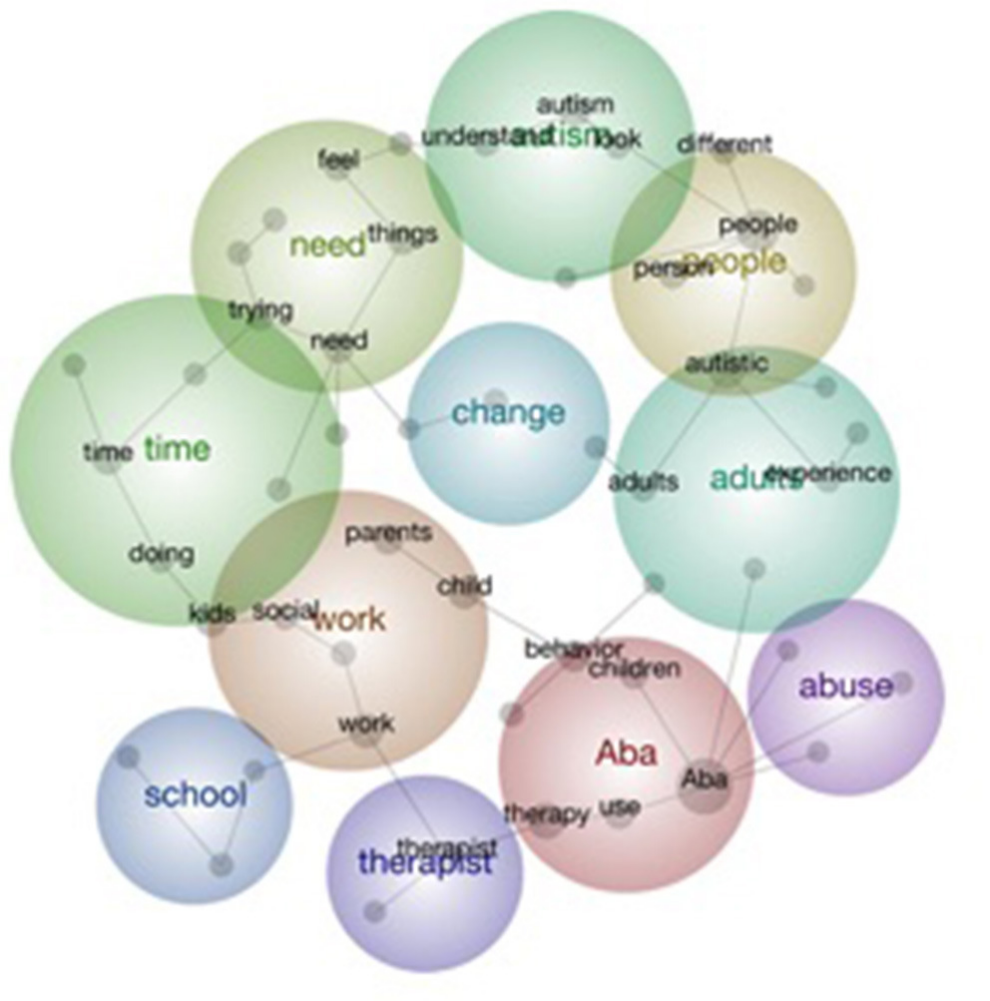
Concept map of open-ended text response using Leximancer software.

The concept map, generated from the topic modeling analysis of all Reddit posts, is presented in Figure 1 provides a birds-eye perspective of the data showing the themes (i.e., bubbles), main concepts (i.e., dots in bubbles), their frequencies, and their interconnectedness. This concept map may be interpreted as users seeking or providing discourse on a specific issue. The concept map suggests that there is limited or no overlap between concepts. On the other hand, there is some overlap with some themes (e.g., work, time, and need), which is expected as they are interconnected. The topic modeling resulted in 11 themes that ranged across various elements, such as autism as a condition and its management (i.e., autism and ABA), stakeholders (i.e., people, adults, and therapists), consequences of ASD, and the support needed (i.e., work, need, school, change, and abuse), suggesting that the discourse around ABA in Reddit is diverse. Table 2 presents the 11 main themes, concepts, frequencies, and examples of meaning units based on the topic modeling. Here, the terms “theme” and “concept” in topic modeling refer to “category” and “sub-category,” respectively, in qualitative content analysis.

- **ABA:** This theme included discussions about definitions, potential, benefits, limitations, and personal experiences. Concepts, such as therapy, behavior, use, and children, were tied together in this theme.
- **Work:** This theme included discussions related to work conducted within healthcare professions or by ABA therapists. Discussions related to whether or not ABA “worked” were included in these discussions. Concepts of kids, child, parents, and social were connected to this theme.
- **People:** Concepts included “autistic,” “person,” and “different.” Discussions in this theme centered around the value of people with ASD and a call for neurodiversity.
- **Need:** The theme refers to whether or not ABA is needed and how much treatment is needed.
- **Autism:** These discussions centered on understanding the nature of autism and the experiences of people associated with autism. Associated concepts included “understand” and “look.”
- **Time:** This theme related to how much time was required for change to occur as a consequence of ABA.
- **Adults:** Discussions in this theme centered around the value of people with ASD and a call for neurodiversity.
- **Therapist:** This theme refers to the role and certification of ABA therapists and their relationship to other professionals.
- **School:** The theme is related to the ability of parents to obtain ABA-based intervention in a school setting and to the education required by ABA therapists.
- **Change:** This refers to both change in behavior or performance and plan or processes associated with ABA intervention.
- **Abuse:** This theme is associated with perceptions of ABA intervention as abusive and creating long-term trauma in individuals who receive ABA intervention.

**Table 2:** Main themes and concepts in discussions around ABA.

**Theme**	**Concepts**	**Frequency (hits)**	**Examples**
ABA	Aba, therapy, behavior, use, children	2,180	I think you're talking about “behavior modification” which is not the same as “applied behavior analysis.” Behavior Modification was used in Gay Conversion “therapy” and Behaviorism is a broad field of psychology that can be applied to training dogs, but ABA is just the applied science of behaviorism, used to effectively treat children with learning disorders:) it's touted as the most effective because it has the most research to back it. There's also a lot of research that ABA is very effective for treating ASD. It's often used school-wide (positive behavior supports) because it's effective for all children.
Work	Work, kids, child, parents, social	1,599	Also, if you claim to work in a hospital, Drs and employees won't tell the patient's parents in their face what level the patient is, but look the medical records of comorbidities. Did you say yoga? By socialization you mean a therapist goes with your child to places right? This ABA thing sounded nothing more than making parents gain some false “social imagination” nugget to convince themselves that their kid will get along with other kids better, at the expense of the kid's overall health.
People	People, autistic, person, different	1,530	That means that autistic people differ a lot between themselves. So even though there are lots of tragic stories online there are also lots of autistic people in the world who are thriving. You are assuming autistic people are like neurotypical people. They are not. We literally have differently wired brains.
Need	Need, things, trying, feel	1,235	I am terrified he may grow up and not be able to express if he is sick or describe how he is sick. These are the kind of reasons I feel like I need to try. They feel set up to fail- and become even more dysregulated, because try as they might in that moment- there's nothing they can do to earn that token/sticker/what have you. Working with dysregulation needs to have strategies to recognize when dysregulation is beginning or may occur, and proactively learning how to remove oneself from those situations.
Autism	Autism, understand, look	892	This is a deep problem in understanding autism. It is, it seems, one of these things that bother normals because it is so fundamental to the human experience to look at the eyes and the eye region. As I got older, this started looking more like my autism was her story to tell, not my own. She'd talk about how she was one of my few advocates when I was young and that she basically had to teach me everything, including how to imagine since my brain wasn't wired for that at first.
Time	Time, doing	638	I typically do 2–4 h sessions but I've gone as long as 11 h a day (although we usually spend a lot of that time doing fun stuff). He's getting clearer and talking much more all the time. He's receiving speech therapy and doing great.
Adults	Adults, experience	408	A quick overview suggests that some number of adult autistics didn't have a good experience with ABA therapy. I don't want to doubt anyone's experience, but plenty of adults who didn't receive ABA seem to associate with this CPTSD label. I think this is just what therapists choose to call autistic style anxiety rather than real PTSD.
Therapist	Therapist	288	Multiple supervisors and other therapists quit before me. I never claimed to be a BCBA, licensed to diagnosed, or anything else I am not. I am a therapist who utilizes ABA to get the most out of my clients.
School	School	200	You provide what we call “behavior assistant.” It's a direct-care position that requires a high school diploma and a 20 h course that covers the very basics of ABA and how to implement some basic procedures. As for ABA, I wonder if there are schools like that in my area. Out of curiosity, how did you find this school?
Change	Change	190	I will then re-evaluate what is going on and make changes if they are necessary. My company provides behavior assistant services as well, and if any of them were to change anything in the plan without my consent they would be fired on the spot. Unfortunately mom changed his meds on him, he went right back to where he was, and had to be placed in a residential hospital. Very sad, I really miss him.
Abuse	Abuse	180	Actually, I do believe it would fall within the definition of emotional abuse. Hitting is not the only form of abuse. My hot take is that “not growing eyebrows” is an allegory for developmental disorders or disabilities or neurodivergence that is then preyed upon by abusive “You's.”

*ABA, applied behavioral analysis*.

### LIWC Analysis

Table 3 presents the mean, SDs, and *t*-test results for 10 key linguistic variables used in the LIWC analysis across four dimensions in ABA-related and baseline Reddit posts. Means reflect the degree to which posts reflect a certain psychological dimension. There was a statistically significant difference between the ABA posts and baseline posts in all of the 10 key variables. The ABA posts had a higher number of words per post. The mean values for authenticity and I-word were higher for baseline Reddit posts. ABA Reddit posts had higher references to others (i.e., social processes) and positive emotions, but less negative emotions when compared to baseline Reddit posts. In addition, ABA posts in Reddit had higher references to health and work, but lower references to home and money when compared to baseline posts.

**Table 3:** Key linguistic analyses variables for Reddit ABA conversations and the baseline Reddit posts.

**Dimension and its description**	**Baseline Reddit: Mean (SD)**	**ABA Reddit: Mean (SD)**	***T*-test results: *t*-value, *p*-value**
**User engagement**			
**Word count:** The number of words that a person used to provide their views and experiences.	35.2 (55.9)	111.4 (155.8)	23.5, <0.001
**Authenticity:** The degree to which a post invokes spontaneity and casualness.	47.4 (38.2)	39.8 (32.5)	−11.3, <0.0001
**I-words:** The degree to which a post invokes an anecdotal or self-referential manner.	5.15 (6.2)	4.3 (4.6)	−8.9, <0.0001
**Social and emotional dimensions**			
**Social processes:** The degree to which a post invokes a social dimension, such as friends or family.	10 (9.2)	12.3 (7.4)	14.9, <0.0001
**Positive emotions:** The degree to which a post expresses positive emotions.	3.7 (5.7)	4.2 (6.9)	3.7, <0.0001
**Negative emotions:** The degree to which a post expresses negative emotions.	2.8 (4.9)	2.4 (3.5)	−4.9, <0.0001
**Biological dimension**			
**Health:** The degree to which a post includes health concepts.	0.68 (0.24)	1.2 (2.2)	12.3, <0.0001
**Personal concerns**			
**Work:** The degree to which a post includes work concepts.	1.8 (3.9)	2.9 (3.3)	15.8, <0.0001
**Home:** The degree to which a post includes home or home life concepts.	0.38 (1.7)	0.19 (0.6)	−14.1, <0.0001
**Money:** The degree to which a post includes money concepts.	0.74 (2.6)	0.32 (2.3)	−8.7, <0.0001

*Mean, SD, and the t-test comparisons are reported. ABA, applied behavioral analysis*.

### LIWC Analyses Across User Categories

#### User Categories Based on Perspectives Toward ABA

ANOVAs were performed to examine the difference in linguistic variables across user categories. The pro- and anti-ABA posts included a higher word count than posts from the undecided/curious group (see Table 4). Group differences were noted on the positive and negative emotion word measures. Pro-ABA and anti-ABA posts included more positive emotion words than the undecided/curious group. The anti-ABA posts included more words weighted with negative emotion than the pro-ABA and undecided/curious posts. Group differences were noted on measures of work, home, and money-related words. The pro-ABA and undecided/curious posts included work-related words with greater frequency than the anti-ABA group. The pro-ABA posts included more words about home life than the other groups. The undecided/curious posts included more money-related words than the other groups. No group differences were noted on measures of authenticity, I-words, social processes, or health.

**Table 4:** LIWC across user categories based on view toward ABA.

**Dimension**	**Mean**			**ANOVA**	**Pairwise comparisons (** ***p*** **-value)**		
	**Pro ABA**	**Anti ABA**	**Undecided**		**Pro vs. anti**	**Pro vs. undecided**	**Anti vs. undecided**
Word count	158.9	161.8	119.6	*F* = 3.7, *p =* 0.024	ns	ns	0.02
Authenticity	39.9	38.9	43.4	*F* = 1.2, *p =* 0.19			
I-words	4.2	3.9	4.2	*F* = 0.7, *p =* 0.48			
Social processes	11.4	11.4	11.0	*F* = 0.4, *p =* 0.67			
Positive emotions	3.4	3.1	2.8	*F* = 4.7, *p =* 0.009	ns	0.015	ns
Negative emotions	1.9	2.9	1.8	*F* = 26.1, *p* < 0.001	<0.001	ns	<0.001
Health	1.3	1.5	1.4	*F* = 2.1, *p =* 0.12			
Work	3.9	2.7	3.5	*F* = 20.3, *p* < 0.001	<0.001		0.004
Home	0.24	0.13	0.17	*F* = 5.5, *p =* 0.005	0.003	ns	ns
Money	0.33	0.24	0.48	*F* = 4.05, *p =* 0.018	ns	ns	0.01

*ABA, applied behavioral analysis; LIWC, Linguistic Inquiry and Word Count*.

#### User Categories Based on Personal Status

ANOVA results suggest that the ASD and professional posts included a higher word count than posts from the parent group (see Table 5). Group differences were noted on the dimension authenticity. The ASD and professional posts used more words weighted with authenticity than the parent group. Group differences were noted on the use of I-words. The ASD posts included more I-words than the other groups. Group differences were noted in social processes. The ASD and professional posts included more words weighted along the social processes dimension than the parent group. Group differences were not noted on the positive emotion dimension but were noted on negative emotion. ASD posts included more negative emotion words than the other groups. Group differences were noted on measures of health and work, but not home and money-related words. Posts from the parent group used more health-related words. Posts from the professional groups used more work-related words.

**Table 5:** LIWC across user categories based on personal status.

**Dimension**	**Mean**			**ANOVA**	**Pairwise comparisons (** ***p*** **-value)**		
	**Parents**	**Professionals**	**ASD**		**Parent vs. professional**	**Parent vs. ASD**	**Professional vs. ASD**
Word count	139.2	176.9	189.6	*F* = 6.3, *p =* 0.002	0.04	0.002	ns
Authenticity	38.1	46.4	50.8	*F* = 15.9, *p* < 0.001	0.001	<0.001	ns
I-words	5.2	5.1	6.6	*F* = 13.3, *p* < 0.001	ns	<0.001	<0.001
Social processes	14.4	10.4	10.8	*F* = 69.3, *p* < 0.001	<0.001	<0.001	ns
Positive emotions	3.6	3.7	3.4	*F* = 0.5, *p =* 0.6			
Negative emotions	1.7	1.9	2.7	*F* = 23.2, *p* < 0.001	ns	<0.001	<0.001
Health	1.6	1.1	1.3	*F* = 9.2, *p* < 0.001	<0.001	0.023	ns
Work	2.7	4.9	2.4	*F* = 86.3, *p* < 0.001	<0.001	ns	0.02
Home	0.27	0.27	0.2	*F* = 1.1, *p =* 0.32			
Money	0.22	0.32	0.25	*F* = 1.7, *p =* 0.18			

*LIWC, Linguistic Inquiry and Word Count*.

## Discussion

This study serves as an initial exploration of discourse among ABA-related posts in Reddit. This paper identified the main themes in online discussions around ABA-based interventions for ASD, examined the linguistic aspects of conversations, and examined the relationship between linguistic aspects and user category. The following highlights the main findings and implications.

### Discourse Themes and Concepts

The most frequent theme (i.e., *ABA*) is not unexpected since posts included in this sample were based on this topic. The themes *work, therapists*, and *school* were identified at varying levels of frequency, but taken together, these themes indicate that in this sample, posters perceive the work of the ABA therapist to be an important conversation. Alternatively, the theme *work* also refers to whether or not ABA-based interventions are effective. A question guiding treatment decisions includes whether or not the treatment is needed and how much treatment is needed (27). *Work* along with the themes *need, time*, and *change* point toward an emphasis on the effects of the treatment. *Work* was also linked with the concept “social.” As noted by Matson et al. (28), a critical question in the literature is whether ABA can be used to modify negative behavior and social skills.

Notably, discussions related to the value of neurodiversity occurred with high frequency (i.e., *people, adult* themes). These posts focused on the need to understand the experience of people with ASD and not diminish the unique contributions of people with ASD. These discussions align with the theme of *abuse*, which is of critical importance to all stakeholders involved in working with people with ASD (12, 29). These posts highlighted concerns that ABA-based intervention has negative long-term consequences on people with ASD. Overall, the themes and concepts suggest a digital landscape that focuses on the effects of ABA-based intervention. Interestingly, the posts did not include themes related to research or evidence-based practice.

### Linguistic Aspects of Conversations

Overall, the comparison of ABA posts and baseline Reddit posts suggests that the language used to discuss ABA is different than the language used in general posts within the Reddit platform. The postings of individuals were focused on personal experiences and opinions as opposed to clinical and research information sharing, which is further represented in the LIWC analysis in that the posts reveal an intimate stance rather than an empirical stance. For example, the high word count in ABA posts is suggestive of high engagement in the topic and complex personal views and experiences. Additionally, posts classified as “authentic” and personal pronouns (i.e., I-words) refer to the individualized experiences rather than broad information sharing. It should be noted that baseline posts had higher means than ABA posts, suggesting that broadly the use of Reddit focuses on personal experiences and/or opinion and may be motivated to signal their position, gain support, or offer support.

It is not surprising that posts were strong in social connections since posts were directed toward intervention, which necessarily includes close personal connections. Emotional responses relate to how people are reacting to a given topic, the degree of immersion in a topic, and the level of agreement about a topic (26). In this sample, positive emotions were weighted more than negative emotions and more than the baseline Reddit posts. Positive emotions may suggest user engagement and alignment with a particular ABA-related topic or the use of civil, polite, and friendly language. The posts with high positive emotion scores included both alignment and amiable language (e.g., *Haha, thanks. It has kind of become my job now- I make videos explaining (autism-related) stuff to people* and *Nice! I will look into this. Thank you. Nice to connect with you*). This relationship between emotional stance, agreement, and immersion is further supported by the word count in that a higher word count is related to higher engagement.

With regard to personal concerns, we examined the concepts of work, home, and money. Words related to work add to the interpretation that the posts were focused on the ABA therapist profession or how ABA worked. It is surprising that more weight was not associated with the sentiments home and money in light of concerns related to insurance coverage related to ABA-based intervention and the impact of ABA-based intervention in the home (30).

### Relationships Between Linguistic Aspects and User Category

#### Comparison Across Views of ABA

Pro- and anti-ABA groups had more word count and positive emotion than the undecided/curious group, suggesting the individuals who had defined positions were more entrenched in the topic. The anti-ABA group had more words weighted with negative emotion than the pro-ABA and undecided/curious groups. The use of negative emotion words is noted within writing about negative or traumatic events (31, 32). The anti-ABA posts may be more likely to include personal negative experiences linked to ABA.

#### Comparison Across Personal Status

Higher word count and use of words along the authenticity dimension in the ASD and professional posts suggest high engagement (i.e., spontaneous talk by making references to self) in the topic as demonstrated through expressing complex perspectives. It may be that parents were more likely to be seeking information related to ABA-based interventions rather than expressing a viewpoint. Additionally, the ASD and professional post use of social process words indicate a sense of connection and relationship with a group. There may be a more defined sense of identify associated within these two groups than may be found in parent groups.

The ASD group included posts with more I-words and negative emotion words than the other groups. As noted by Kapp et al. (15) and McGill and Robinson (16), adults with ASD often report negative experiences associated with ABA-based intervention. Taken together, it may be that individuals with ASD were more likely to express psychological states related to their experiences and perspectives.

### Implications for Practice

Understanding the nature of information shared online may help healthcare professionals support families in evidence-based decision-making. These data illustrate that much of the information shared centers on personal information and/or opinion. Posts include diverse topics, such as benefits and the limitation of ABA-based intervention, call for neurodiversity, and the role of the ABA therapist. Engaging in conversation with families, asking questions, and opening the dialogue around these topics may be helpful in understanding their stance and providing individualized guidance. Being prepared with accessible evidence-based information may help healthcare professionals dispel misinformation.

### Strengths, Limitation, and Future Directions

The topic modeling and linguistic analysis provided a broad understanding of the data (i.e., landscape the discourse) rather than specific discussions. While the automatic process has the advantage on saving time, it is also limited in its ability to provide in-depth analysis. For example, the theme “work” included posts that referred to work as in “it can work” and work as a “job.” In this context, the same word or concepts have different meanings, which the software does not differentiate. The study also used a word counting approach to linguistic analysis, which ignored the context and intended audience. That said, this simple word counting approach does provide surprisingly clear and reliable insights into a person's psychology (25).

It is important to note that the data may not be representative of the general population, which is likely the case for most social media studies. For example, Reddit users have been found to be predominantly male and younger (under 30 years) (33). The users are anonymous and not many details are known about the population. Although we anticipated that the users of this community included parents of children with ASD, health professionals with different views toward ABA therapy, and individuals with ASD, we could not confirm the role. Additionally, we do not know the diversity of the sample with regard to race and ethnicity. While not knowing the user demographics is a limitation, the anonymous nature of Reddit is likely to produce a more truthful response (or ecologically valid data) (34). Finally, the context in which the posts occurred is difficult to examine, which limits the interpretation of the posts. The total number of posts on this topic is limited, which makes it a very specialized discussion relative to the volume of discussions occurring on Reddit.

Future studies should focus on performing more in-depth analysis of ABA discussion to examine the specific narratives used and the tensions among posts from these groups. Moreover, in the current study, the key dimensions and the generic LIWC dictionary were used for analysis, but future studies should aim to develop and use concepts and dictionaries specific to ASD.

## Data Availability Statement

The raw data supporting the conclusions of this article will be made available by the authors, without undue reservation.

## Author Contributions

All authors listed have made a substantial, direct, and intellectual contribution to the work and approved it for publication.

## Conflict of Interest

The authors declare that the research was conducted in the absence of any commercial or financial relationships that could be construed as a potential conflict of interest.

## Publisher's Note

All claims expressed in this article are solely those of the authors and do not necessarily represent those of their affiliated organizations, or those of the publisher, the editors and the reviewers. Any product that may be evaluated in this article, or claim that may be made by its manufacturer, is not guaranteed or endorsed by the publisher.
